# Patients' Perspectives on Attributes While Choosing Minimally Invasive Surgery for Benign Prostatic Hyperplasia Procedures: Experience from Men Undergoing Water Vapor Thermal Therapy

**DOI:** 10.1089/end.2022.0607

**Published:** 2023-05-15

**Authors:** Sirikan Rojanasarot, Ben Cutone, Kathryn Durand, Kevin C. Zorn, Bilal Chughtai, Naeem Bhojani, Dean S. Elterman

**Affiliations:** ^1^ Health Economics & Market Access, Boston Scientific, Marlborough, Massachusetts, USA.; ^2^ Division of Urology, University of Montreal, Montreal, Canada.; ^3^ Department of Urology, Weill Cornell Medicine, New York, New York, USA.; ^4^ Division of Urology, University Health Network (UHN), University of Toronto, Toronto, Canada.

**Keywords:** benign prostatic hyperplasia, water vapor thermal therapy, patient survey research, interviews, lower urinary tract symptoms, minimally invasive surgical therapy

## Abstract

**Objective:**

To understand which attributes men with benign prostatic hyperplasia (BPH) undergoing water vapor thermal therapy (WVTT) find important while considering treatment options for the condition.

**Methods:**

Men (*n* = 170) with lower urinary tract symptoms due to BPH who underwent WVTT between April 2019 and November 2020 in a Toronto urologic clinic were invited to participate in an online survey. The survey included eight attributes of BPH surgical procedures and five attributes of WVTT. Patients were asked how important each attribute was to them before they selected a BPH procedure and decided to undergo WVTT.

**Results:**

In total, 128 respondents (75%) completed the survey. A majority of the respondents were White (88%), married (83%), and aged 60–69 years old (45%). Approximately 97% of respondents rated the ability to avoid further BPH treatments as “very important” or “extremely important,” followed by duration to return to normal activities (79%), and wait times to receive the procedure (57%). Only 47% of patients reported that postprocedural catheterization was important. For WVTT, 98% of the respondents rated avoiding more invasive surgical treatments and 88% rated a quick recovery as important attributes.

**Conclusions:**

Among men with moderate-to-severe BPH undergoing WVTT, the most important attributes for selecting a BPH surgical procedure were avoiding further BPH treatments, returning quickly to normal activities, and reducing treatment wait times. Most men chose WVTT to avoid more invasive procedures and have a quick recovery.

## Introduction

A wide range of treatment options exists for the management of lower urinary tract symptoms (LUTS) due to benign prostatic hyperplasia (BPH), and depending on the severity of the disease, includes medications, surgery, and minimally invasive therapies.^1^ Although drug therapy is considered the dominant first-line treatment for mild-to-moderate BPH, adverse events such as dizziness, erectile dysfunction, and decreased libido contribute to low patient adherence rates and frequent therapy discontinuation.^2^ For men with BPH who do not respond to drug therapy or cannot tolerate drugs, urologists may recommend surgical procedures.^3^

The surgical management of LUTS due to BPH ranges from minimally to more invasive surgical procedures. Many of the most common surgical procedures, such as transurethral resection of the prostate (TURP), are performed in an operating suite with general or spinal anesthesia and are considered invasive for the patient.^4^ More invasive surgical procedures have the potential for a longer recovery period after surgery and have been associated with a substantial risk of both ejaculatory and erectile dysfunction.^5^ In addition, if the procedure is not durable, patients may require retreatment. Water vapor thermal therapy (WVTT) is a minimally invasive surgical procedure for BPH, in which a few drops of heated water are used to treat the excess prostate tissue causing BPH symptoms. Current evidence from real-world settings and randomized clinical trials shows that WVTT therapy provides an efficient, safe, and effective treatment option for the relief of moderate-to-severe LUTS caused by BPH.^6,7^

Given the differences in the clinical and economic outcomes, the surgical options to manage LUTS due to BPH have different treatment profiles and attributes. In addition, clinical guidelines, such as the American Urological Association (AUA) guidelines, recommend shared decision-making between patients and providers in treatment selection.^1^ Therefore, it is critical to understand the most important attributes that patients consider when selecting treatment options for BPH. This knowledge can inform providers to make appropriate treatment selections for their patients, potentially leading to higher patient satisfaction.^1^ The objectives of this study were (1) to identify the most important attributes for selecting BPH surgical procedures from the perspective of men with moderate-to-severe BPH who had previously received WVTT and (2) to determine the most important attributes of the WVTT procedure before the patients decide to undergo the treatment.

## Materials and Methods

### Study population

This study received Institutional Review Board (IRB) approval from the Research Ethics Committee of the University of Toronto. All respondents provided written consent to participate in the clinical surveys and signed a written consent form. All men with moderate-to-severe LUTS due to BPH who were surgically naive and underwent WVTT between April 2019 and November 2020 in a urologic clinic in Toronto, Canada, were invited to participate in an online, cross-sectional survey (*N* = 170). All men were counseled preoperatively by the treating urologist regarding the effectiveness, durability, and risks of all surgical BPH options available in Canada, including TURP, photoselective vaporization of the prostate, aquablation therapy, temporarily implanted nitinol device (iTind), WVTT, and medical therapy.

Anatomical endoscopic enucleation of the prostate was not discussed during the treatment selection due to the limited uptake of the therapy in Canada.^8^ The final BPH surgical treatment option was determined by shared decision-making between the urologist and individual patient.

### Survey design and administration

A survey of closed and open-ended questions was created using Microsoft Forms (Redmond, WA, USA), following a previously published guideline for survey construction.^9^ The survey explored patient demographics, eight attributes for selecting a BPH surgical procedure, and five attributes associated with the WVTT procedure during the past 8 months (Table 1). The attributes associated with BPH surgical procedures and WVTT were identified from a literature review and validated by a BPH clinical expert. The survey was piloted to ensure that all questions were appropriately structured and revised based on respondents' feedback.

**Table 1.** Survey Composition on Patient Perceptions of Benign Prostatic Hyperplasia Surgical Procedures

Eight attributes for selecting a BPH procedure	Five attributes associated with the WVTT procedure
• Procedure performed in a nonhospital setting • Procedure length of time • Length of time to return to normal activities after the procedure • Catheterization after the procedure is required • Wait times to receive the procedure • Procedure is performed under sedation or local anesthesia without the need for general anesthesia • BPH treatment for recurring BPH symptoms after treatment (treatment durability) • Cost of the procedure to the patient	• Preserve sexual function • Avoid taking daily BPH medications • Avoid more invasive surgical treatments, that is, TURP • Avoid long wait times to receive other surgical treatments for BPH in the public system • Quick recovery and return to normal activities

BPH = benign prostatic hyperplasia; TURP = transurethral resection of the prostate; WVTT = water vapor thermal therapy.

Using a 5-point Likert scale (1: not important to 5: extremely important), patients rated eight attributes for selecting a BPH procedure, including procedure wait times, length of time to return to normal activities postprocedure, and the ability to avoid further BPH treatments. Based on their experience with the WVTT procedure, patients used a 5-point Likert scale to rate the importance of five attributes associated with the WVTT procedure, including preserving sexual function, avoiding more invasive surgical treatments, quick recovery, and returning to normal activities.

A structured survey was administered to all eligible patients between February 10, 2021, and March 3, 2021. To ensure the highest response rates, a reminder was sent 2 weeks after the initial invite. Survey responses were tabulated for each attribute. All data were presented descriptively as a crude number and percentage of respondents per question. In addition to the analysis of the overall patient cohort, subgroup analyses by age groups were conducted to explore the age-group preference classification. The subgroup analyses were reported as the percentages of respondents who rated “very important” or “extremely important” for each attribute from the eight attributes for selecting a BPH surgical procedure and five attributes associated with the WVTT procedure.

## Results

Of the 170 men invited to participate, 128 completed the survey, reflecting a 75% response rate. The majority of the respondents were White (103/117, 88%), married (100/121, 83%), and aged 60–69 years (58/128, 45%) (Table 2).

**Table 2.** Patient Characteristics of Survey Respondents (*n* = 128)

Characteristics	Proportion
Age group (*n* = 128)	
<48	0/128 (0%)
48–59	22/128 (17%)
60–69	58/128 (45%)
70–79	40/128 (31%)
80+	8/128 (6%)
Race (*n* = 117)	
White	103/117 (88%)
Black	2/117 (2%)
Other	12/117 (10%)
Marital status (*n* = 121)	
Single	10/121 (8%)
Married	100/121 (83%)
Separated	5/121 (4%)
Divorced	6/121 (5%)
Employment status (*n* = 124)	
Full-time	36/124 (29%)
Part-time	9/124 (7%)
Self-employed	19/124 (15%)
Unemployed	3/124 (2%)
Retired	57/124 (46%)
Gross household income (*n* = 128)	
Under $100,000	44/128 (34%)
$100,000–$249,999	51/128 (40%)
$250,000+	33/128 (26%)

**FIG. 1.**
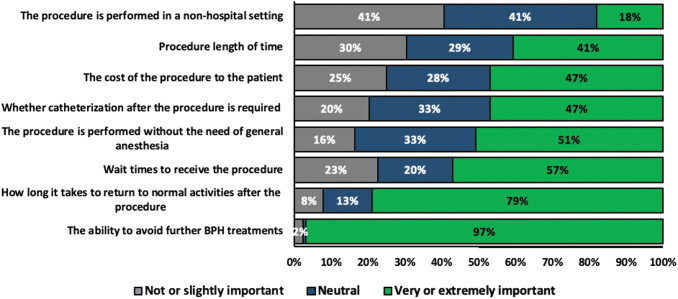
Patient rating of surgical attributes before selecting a BPH procedure. BPH = benign prostatic hyperplasia.

**FIG. 2.**
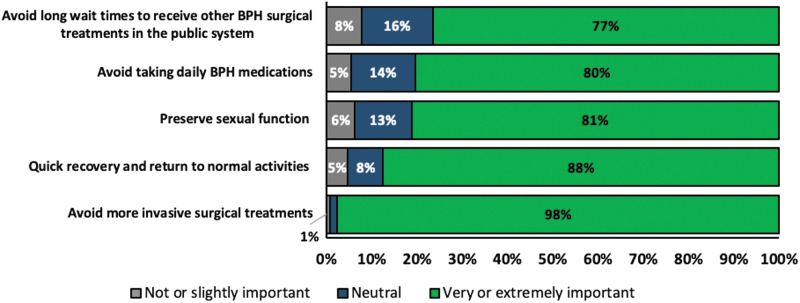
Patient rating of attributes associated with the water vapor thermal therapy procedure.

Approximately 97% of respondents rated the ability to avoid further BPH treatments as a “very important” or “extremely important” attribute when selecting a procedure, followed by duration to return to normal activities (79%), reduced procedure wait times (57%), and avoiding a procedure that required general anesthesia (51%) (Fig. 1). Only 47% of the respondents reported that postprocedural catheterization was an important attribute in selecting a BPH procedure (Fig. 1). For WVTT, 98% of the respondents rated avoiding more invasive surgical treatments as a “very important” or “extremely important” attribute and 88% rated a quick recovery as a “very important” or “extremely important” attribute (Fig. 2).

For age-group analyses, nearly all men aged 48–59, 60–69, and 70–79 years rated the ability to avoid further BPH treatments as “very important” or “extremely important” while 100% men aged at least 80 years rated how long it takes to return to normal activities after the procedure as “very important” or “extremely important” (Supplementary Fig. S1). The results are consistent with the attributes associated with the WVTT procedure since avoiding more invasive surgical treatments is the most important attribute across all age groups (Supplementary Fig. S2).

## Discussion

The results of this study showed that Canadian men with moderate-to-severe LUTS preferred BPH surgical procedures that allowed them to minimize procedure wait times (57%), avoid anesthesia (51%), quickly return to normal activities (79%), and avoid further BPH treatments (97%). Avoiding further BPH treatments was deemed the most important attribute by a majority (97%) of the respondents. Retreatment rates are an important consideration in the evaluation of BPH surgical treatment options and vary substantially between invasive and minimally invasive surgical procedures.^10–12^ Reoperation rates for transurethral needle ablation and transurethral microwave thermotherapy at 5 years have been reported to be as high as 14% and 9%, respectively. However, the 5-year retreatment rate for the minimally invasive WVTT procedure has been estimated to be between 4% and 5%, as shown in both clinical and real-world trials.^10–12^

Some treatment modalities may offer lower retreatment rates but with a higher degree of invasiveness and a negative impact on sexual function.^1^ Given the importance of this attribute from the patient's perspective, it is vital for health care providers to inform patients about the retreatment rates across various BPH surgical options.

In this study, 47% of respondents reported postprocedural catheterization as a “very important” or “extremely important” attribute for selecting a BPH procedure. While catheterization may be bothersome to the patient, respondents weighed long-term outcomes, such as reduced retreatment rates, to be more important than short-term care considerations. This finding is consistent with other studies showing that men with BPH are often more worried about the long-term risks of their underlying BPH rather than short-term outcomes such as immediate symptom relief.^14^ Our study found that 81% of respondents value preserving their sexual function. Protection of sexual function may have driven nearly all respondents' preference (98%) to rate avoiding more invasive surgical procedures as a “very important” or “extremely important” attribute in their consideration of a WVTT procedure. Given that safe and effective minimally invasive treatments are available, patients may prefer not to undergo invasive procedures because of concerns regarding associated adverse events.^5^

Several treatment options exist on the continuum of care for BPH management, ranging from medical management to invasive surgery. These treatment options vary substantially by several characteristics such as invasiveness, efficacy, safety, side effects, and durability,^1^ as well as costs and cost-effectivness.^14^ Clinical decision-making regarding the choice of BPH therapy has typically been informed by the patient's severity of symptoms, gland size, and anatomical features. In many cases, there has been a disconnect between physicians' recommendations for BPH treatments and men's preferences for BPH treatments. A systematic review of seven studies (*n* = 1851 patients) examining physician and patient preferences for selecting an appropriate BPH treatment found great variability between patient and provider preferences.^14^

A US national telephone survey by Kaplan et al. examined the preferences and attitudes toward BPH treatments among patients and providers (*n* = 419 patients; *n* = 100 urologists; *n* = 100 primary care physicians).^15^ The study found that providers believed that their patients were more concerned with immediate symptom relief, while their patients expressed greater concern with the long-term risks of BPH.^15^ Similarly, a UK interview-based survey by Watson et al. (*n* = 208 men) found that patients preferred therapies affecting long-term BPH disease progression over those that provided short-term symptom improvement.^16^ The results of these studies demonstrate that physicians may have contrasting views to their patients regarding BPH treatments and may not fully appreciate the patient's perspective when selecting an appropriate therapy.^14^

Patient therapy selection should rely on a careful physical evaluation as well as an informed discussion between the provider and patient regarding the different attributes and risk/benefit profiles of each treatment option.^17^ Recently, a web-based decision aid integrated patient preferences for BPH therapies, which strengthened the discussion of treatment options between patients and their providers.^17,18^ Such a discussion may necessitate a referral to another clinician for the chosen treatment.^1^ The results of the present study as well as recommendations from the AUA underscore the importance of including the patient's perspective in the selection of appropriate BPH treatment as part of a *shared* decision-making approach between patients and providers.^1^

### Limitations and strengths

The present study population included men undergoing a WVTT procedure at a single clinic without including men who decided to undergo a different surgical treatment due to the limited sample size among those men, which may limit generalizability and transportability. The results may be different among men undergoing a different type of minimally invasive surgery. Future studies could include men who decided to undergo a different treatment option from multiple sites, as well as conduct comparative analyses of attributes from different surgical procedures to better understand comparative patient preferences. Patient perceptions of BPH surgical attributes may differ based on geographic differences in surgical practice patterns (i.e., wait times, use of general anesthesia), differences in procedure costs, differences in care setting (i.e., hospital *vs* nonhospital setting), and differences in surgical procedures offered by other community urologists.

This study examined patient perceptions; other patient outcomes, such as ejaculation preservation, International Prostate Symptom Score (IPSS), International Index of Erectile Function (IIEF), complications, and quality of life, were not analyzed. This study examined how patient perceptions influenced treatment choice but did not address whether WVTT was the best treatment choice for each patient. Patients undergoing WVTT may have been informed about the benefits and risks of WVTT preprocedure, potentially resulting in different ratings for these attributes. Finally, as patients were asked to evaluate their experience retrospectively, recall bias may have either underestimated or overestimated the true effects or associations of the domains being measured.^19^

This study has several strengths. This is the first study to evaluate the most important attributes of WVTT from the patient perspective, revealing the “patient's voice” in the selection of BPH therapies. These findings highlight the importance of “patient-centeredness” in BPH, which positively affects a patient's quality of life. Achieving a better understanding of important attributes from the patient's perspective may help manage patient expectations and increase patient satisfaction. Providers can use these findings to guide conversations with patients and set their expectations before they select and undergo a BPH procedure. This study had a high response rate (75%) compared with the average response rate of web-based surveys (46%) on patient outcomes.^20^ While the survey tool was not validated, piloting the survey tool and revising it based on respondents' feedback enhanced the tool before survey administration.

In addition to clinical and economic attributes considered in this study, patient price preferences and willingness to pay for a therapy are also another important patient valuation, which has been well-studied in other prostate conditions, including prostate cancer.^21^ Future research should examine willingness to pay for invasive and minimally invasive BPH procedures, given the different attributes of each type of procedure (i.e., cost, procedure length, setting of care, recovery time, and durability).

## Conclusions

The results of this study showed that the most important attributes for choosing a BPH surgical procedure were minimizing procedure wait times, avoiding anesthesia, quickly returning to normal activities, and avoiding further BPH treatments. Postprocedural catheterization was not a leading factor in men when choosing a BPH procedure. Most men chose WVTT to avoid more invasive procedures and recover quickly. WVTT provides many attributes that are important to men when choosing a BPH surgical procedure.

## Supplementary Material

Supplemental data

Supplemental data

## Abbreviations Used

AUA: American Urological Association
BPH: benign prostatic hyperplasia
LUTS: lower urinary tract symptoms
TURP: transurethral resection of the prostate
WVTT: water vapor thermal therapy
