# Supplementary material for: Patients' Perspectives on Attributes While Choosing Minimally Invasive Surgery for Benign Prostatic Hyperplasia Procedures: Experience from Men Undergoing Water Vapor Thermal Therapy
Source: J Endourol. 2023 May 15;37(5):575–80. doi: 10.1089/end.2022.0607 (PMC10210213; doi:10.1089/end.2022.0607)

Supplementary Figure 2. The Percentage of Men From Different Age Groups Rating Each Attribute from the Five Attributes Associated with Water Vapor Thermal Therapy Procedure as Important


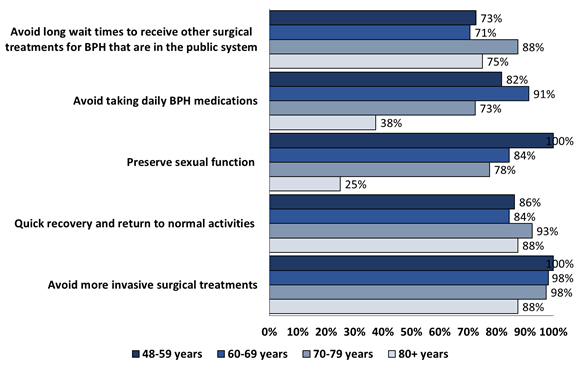

Supplement: Supplemental data [file Suppl_FigS2.docx]
